# Academy of Stomatology

**Published:** 1895-06

**Authors:** 


### ACADEMY OF STOMATOLOGY.

THE regular monthly meeting of the Academy was held at the rooms April 23, 1895, the President, Dr. Jack, in the chair.

The paper of the evening, entitled "The Scientific Spirit and the Ethics of Dental Practice," was then read by Dr. Chas. J. Essig.

(For Dr. Essig's paper, see page 343.)

### DISCUSSION.

*Dr. Roberts.*—I think I can see one way by which there may be a very great difference of opinion in the number of cavities to be found in a mouth. I went into an office some time ago and the dentist had a patient of mine in the chair. He said, "I have examined the patient's mouth and put in four gold fillings, and there are about two more and her mouth will be in perfect order." I looked at the instrument he was using and found it was quite a large excavator. I took a probe and found twelve cavities which I demonstrated to him and her; well marked cavities in the crowns of the teeth and the proximal surfaces.

The essayist made a statement that gold is recognized as the best material for filling teeth,—is that so?

*Dr. Essig.*—I said it is so regarded; that is not my opinion.

*Dr. Roberts.*—I intended taking exception to it as being the best under certain conditions.

*Dr. Grosser.*—I think in Dr. Roberts's case there was no difference of opinion; it was a matter of lack of ability to diagnosticate. I suppose all are acquainted with the kind of enamel in the so-called fissures, in which there has been imperfect development, and where without experience it would certainly be decided decay was not present; so that in the case mentioned it was a mere lack of ability.

*Dr. Currie.*—I think often we are puzzled to know just what the code of ethics would require of a man when a patient presents himself for examination, and we cannot help but discover cavities in the mouth. I recently had a patient where I not only found simple cavities, but some in which the pulp was very nearly exposed. She assured me that in the last few days, not over five, she had been to her dentist, and he had been unable to find any cavities. Now, just what is expected of a man in that position I do not know. Surely our profession requires of us that we tell the truth, and our patient demands our best judgment.

I must confess that while there are some differences of opinion which might be ascribed to judgment, yet there are undoubtedly some which require criticism. Does the essayist think those cases should be treated that way, or should we pass them by and permit our charity to cover a multitude of unsightly things?

*Dr. Essig.*—It seems to be a case so very clear that a few words will answer it. It is surely the duty of any dentist in whose hands a patient comes to find cavities, if they are there; but when criticising the other dentist is attempted, it shows a low standard of ethics, and a man who is a gentleman would not do it.

*Dr. Roberts.*—Is not the fact of finding cavities a criticism of the other dentist? What are you to do, refer the patient to the former dentist?

Dr. McQuillen said that all were likely to make mistakes, and a dentist would sometimes find that, upon a patient returning, he had missed something, and he was honest enough to say it had been so in his case. The doctor spoke of a maxim of his father of which he often thought, and that was, if he had not a good thing to say about a man, he did not say anything at all. He thought that was as good a code of ethics as a man could work under in his profession.

*Dr. Roberts.*—Will Dr. Essig give an opinion on the following case. A patient of mine brought her sister to me, who had just been under the hands of another dentist, to have her mouth examined. Two incisors had been filled, the upper one with gold, and in one of them there was a large space between the gold and the enamel of the tooth. I could pass the excavator directly into it. Her sister was watching at the time, and said, "What is that?" I said, "I think the doctor has slipped a little here, and I would advise her to return and ask him if it was all right." Is that the right thing to do?

*Dr. Essig.*—I think the sending a patient back to a previous dentist is right; that is exactly what I would do myself. After that, if the patient comes to you, you are free. But to openly criticise to the patient the work of another dentist is a thing that I cannot characterize in strong enough terms.

*Dr. Christensen.*—The essayist said that no amount of education would make a gentleman out of a man who was not one by nature; it seems to me that is really so. Persons are taken into the colleges in this country with but a limited amount of previous training; this is the great difference between this and the old country. I do not mean to hold the old country up as an example of something better; we do on the other side too much of the general training or education, and regard special education too little; but it seems to me the general education should not be neglected. I think there ought to be a standard established before a college is entered.

Dr. Essig was of the opinion that a man, as he stated in his paper, would never be a gentleman unless he was one by nature or by training, or rather, that no written code would create that which had no existence.

*Dr. Deane.*—I was thinking of some points in active practice, where no code of ethics would exactly cover the conditions. There are many such, especially in the treatment of children's teeth, in which it would not do to operate at that sitting, where it would be better to do so at a later period; also when the patient passes into the hands of one who is unscrupulous, who will force an excavator into the fillings and say, "There is a cavity." That person ought, in my opinion, to be ruled out of any society or association.

I am, perhaps, bitter on this point, for the reason that lately I have had a law suit. I had filled the front teeth with phosphate, two approximal cavities in the incisors, because I felt the tooth substance was too weak to stand anything in the way of gold. The young lady claimed on the witness stand that she went to Baltimore and a dentist there filled them with gold and they had gold fillings in at the time. Fortunately for me, she claimed that all of my work had to be taken out and refilled with gold, the back teeth as well as the front. The two experts I had with me examined the mouth and found that all the amalgam fillings were still in the posterior teeth and she had no decay in them. I am still of the opinion that the phosphate would have served equally as well as gold and perhaps better for a few years. It is such men that the code of ethics should reach in some way.

*Dr. Roberts.*—I did not finish the description of the case I referred to. I said to the patient, "Will you take a note back to your doctor," who happened to be a friend of mine, as well as her dentist. She replied she would, but changed her mind subsequently. She stated she would write him a note, if I did not object, and tell him that she had left his office, and would seek services elsewhere. I would like to know just what is required of a man in a condition of that kind.

*Dr. Burchard.*—Some nineteen hundred years ago, I think there was a code of morals given to the world that will cover all points brought out this evening, or can possibly be brought up at any time; and it is comprised in what is ordinarily known as the Golden Rule. Rudyard Kipling, in one of his works, speaks of a certain ape, and says it has too much ego in its make-up, and that is a disease that afflicts not only apes but a majority of mankind; and a lesson man needs to learn is that ego and alter-ego are not enemies, but brothers; that what the world needs is the development of more of the altruistic spirit, and less of the egoistic spirit; by that I think all differences of this sort will be solved.

*Dr. W. H. Trueman.*—I am very much impressed with the paper; it is a very good one on a subject not very often considered, but which I think might be discussed very profitably. It indicates a deplorable factor in the profession, and I should think it was overdrawn; at least, I should hope so. I have met with a few instances of that kind; patients have left me and have left the other man.

How shall we get at this matter and remedy it? That is the main point. Professor McQuillen struck the nail on the head many years ago when he said a gentleman does not need a code of ethics, and a man who is not a gentleman will not heed it. If we would find the remedy in the matter, it seems to me we ought to go to our dental societies, beginning at the one standing on top, for I think we find the code of ethics is there rather more rudely broken than in any cases Professor Essig has related to us.

I have had some little experience of that kind,—I am not growling. I was at the meeting in Cincinnati some years ago, and an excursion was arranged for the afternoon to the Zoological Garden, and those participating were to be supplied with badges. I went to the one in charge and asked for one, and he said they were for members only. I had paid my dues and had the ticket in my pocket and showed it to him. He said, "How do I know you are the man on that ticket; how do I know but that you picked the ticket from the floor; you must be identified by some one." A prominent member passed by, and I said, "He knows me." He looked at me and said, "Doctor, you had better be identified by a member of your local society." I felt hurt. I am inclined to think that it was not the right kind of treatment. I had been chairman previously of a section and had read a paper. I think this was a greater violation of the code of ethics than any Dr. Essig has mentioned.

On another occasion I was requested to prepare a paper, which I did at considerable expense and time, and sent it in and received a very complimentary letter. I did not attend the meeting, but the paper was read, and some one called out, "A barefaced advertisement," or words to that effect. Yet I was not there as a salesman and had nothing to sell. I had no interest except that if I find a good thing I desire my professional brethren to share it with me. The next communication I had with the society was my resignation.

I think these things ought not to be, and the code of ethics should be lived up to in a kindly spirit in our dental societies.

There is another point that I think is a violation of the code of ethics. We all know we have honest differences of opinion; you very often find a practical paper on some practical subject, in which some new process is broached, and the writer generally begins by finding fault with every one else and every other method except the one he has adopted. Sometimes very important improvements are made. I remember one instance at Asbury Park, where a paper was read.

Remarks were made which were very uncalled for and were a gross violation of the code of ethics. All professional men who are original thinkers will at times feel differently and think differently, and we should respect each other's opinions if a new thing is broached; we cannot always adopt it at once, needing time to consider it. It appears to me that when we get to that point in our dental societies where we can discuss differences of opinion kindly, we shall have gone a long way in correcting these matters to which Professor Essig has referred.

*Dr. James Truman.*—I do not feel I have very much to say on this paper. It is a timely one, for I think there is altogether too much violation of the code not only of that described by Dr. William Trueman in societies, but in individuals. I can have but little sympathy with it. Gentlemen will never do that kind of thing; but I doubt whether education will help very much; it cannot eliminate inherited proclivities.

I happened to be present when the paper to which Dr. Trueman has alluded to was read. It was the American Dental Association. He didn't give the name, but I give it. I was shocked that any one should be so treated in the leading association of the country. I suppose that somebody was tired; worn out by a long session, and coarsely made that remark. It was unpleasant and uncalled for.

But it seems to me there is a deeper thought in this matter, and that is that men differ and may differ honestly in regard to practice. They may say, for instance, there is not a cavity in this mouth that needs filling, and then the patient may visit another man, and he may pick out twenty or thirty in the same mouth. Why? Simply because this last operator lacks experience. Let me illustrate this. Take a person with very dense teeth, over forty years of age. Now suppose that patient falls into the hands of another who has probably just graduated. I do not care from what school; it is immaterial. With his glass he will observe the black lines running over the teeth where the fissures formerly were, and he will say, "Why, you have fifteen or twenty fillings needed on the masticating surfaces of the teeth." Let this patient fall in the hands of an experienced operator and he will say to that individual, "You have no cavities that need filling." His judgment would be that there had been an arrest of decay there and that there would never be a necessity for filling the cavities, and to cut out these dark lines would not only be a waste of time and strength of a man, but a wrong done. The differences of opinion here may be honest; inexperience against experience.

Again, there is a difference with many of those who are advanced in the profession. For instance, very recently in a discussion of Dr. Perry's paper, I made some remarks endorsing the views held. I received a private letter taking me severely to task for the opinion expressed, the writer stating that he was surprised that I would advocate the idea that filling of children's teeth with gold was wrong practice; that he had filled teeth for many years, and had been trained under me, etc., and felt almost hurt that I should now advocate filling teeth with gutta-percha. Here was an honest difference of opinion, and there must be some reason for it. I cannot conceive how two men, equally instructed, should arrive at such opposite conclusions. I am inclined to attribute it to climatic conditions: that my friend and critic can do in the Sandwich Islands that which is not possible for us to do here, with our ever-changing temperature. So, therefore, the more we look at the matter outside of personal antagonisms, I feel that there is still a wide margin for an honest difference of opinion, and while it is not necessary to say anything derogatory of our brethren, we must still give an honest judgment on cases presenting.

*Dr. Essig.*—If it is in order for me to rise again, I would like to speak on some points Dr. Truman brought out. Patients do quote us sometimes, and quote us probably without permission. I notice that of the new patients who come to my hands since I have been in practice, particularly in the last ten years, they will not mention any names; and there is no necessity for criticising other dentists, whoever they may be. Sometimes patients will quote our remarks to other dentists, and that will make bad feeling. Now, I want to mention, as bearing on this point, a case that occurred to me five or six years ago. A stranger came to my office early in September, after the vacation was over. He explained to me that he was a lawyer and had had a partial denture made, and that it interfered with speaking; that he spoke in court almost every day, and it was essential for him to have clear articulation. He exhibited the piece to me and explained just how it behaved; that it was a little loose on the inner side near the edge of the plate; and said that he had trouble in forming words ending with s, and had difficulty sometimes to make himself understood, particularly in speaking rapidly. He gave me the name of the dentist who made it, and I told him at once to go back to him and tell him just what he had said to me, and I had no doubt he would make it right, as I declined to touch it. In two or three weeks he came back and said he had done as I wished, but that the dentist said nothing else could be done; and he was not willing to do anything more, and that he had quit with him, and as far as he was concerned the transaction was at an end. I did not like to take up the case at all, but the man was persistent. The name he gave me was that of an acquaintance, and I thought I would communicate with him before doing anything for the man. In a few days I received a letter from the dentist, stating that the first piece of work had not been paid for, and among other things stated that the patient had said to him that I said the work was unscientific. Now, it is a word I never should have used in connection with a denture. He simply invented it. He paid my bill, and I instructed my secretary that whenever that man called to refuse to give him an appointment. That was a case where a man had gratuitously quoted me falsely.

*Dr. Huey.*—There is one phase that has not been spoken of. Most of us seem to think the Golden Rule requires us to do unto other dentists as we would have them do to us, but how about the patient? I think the patients have to be considered in this matter as well as the dentists. I especially think of a little experience of a patient who had been in my charge, fifteen or sixteen years of age, having pyorrhœa, and when she came to me just after her marriage a few years later, she was in a most deplorable condition. I had her mouth in fairly good condition, when she went to Europe; she returned a few weeks since and certainly three of the teeth will have to be lost. She left her children on the other side. She asked the cause of the condition, and I told her it was because the tartar had not been removed from them. You sometimes have to criticise even your professional brethren.

*Dr. Guilford.*—Dr. Huey spoke of the duty we owe patients, and I think it is a very important matter. In most of what has been said it has been from the stand-point of the dentist; that is all right, but we want to consider the patient also. I had a case come to me not long ago, of a lady from another city, having had work done by a dentist there whom I knew very well. She wanted me to look over the teeth and see if anything was needed, and I found at least half a dozen cavities, some of them half-way into the pulp, and that could have been filled easily a year before. I did not say anything about the dentist, but let her draw her own inferences. There is no use of trying to uphold another dentist when he has been careless or done wrong.

Dr. Gaskill presented a plaster cast to the association.

Dr. Essig stated that, in accordance with his promise at a previous meeting, he had sent to the society the transactions of the New York Odontological Society from 1876 to 1892. Also the transactions of the Pennsylvania Society from 1879 to 1883. One or two years of the New York transactions are missing, which it would be well for the members to supply, if possible.

Adjourned.

